# Cryoelectron Microscopy Structures of AdeB Illuminate Mechanisms of Simultaneous Binding and Exporting of Substrates

**DOI:** 10.1128/mBio.03690-20

**Published:** 2021-02-23

**Authors:** Christopher E. Morgan, Przemyslaw Glaza, Inga V. Leus, Anhthu Trinh, Chih-Chia Su, Meng Cui, Helen I. Zgurskaya, Edward W. Yu

**Affiliations:** a Department of Pharmacology, Case Western Reserve University School of Medicine, Cleveland, Ohio, USA; b Department of Chemistry and Biochemistry, University of Oklahoma, Norman, Oklahoma, USA; c Department of Pharmaceutical Sciences, Northeastern University School of Pharmacy, Boston, Massachusetts, USA

**Keywords:** *Acinetobacter baumannii*, AdeB multidrug efflux pump, cryo-EM, multidrug resistance, structural biology

## Abstract

Acinetobacter baumannii is a Gram-negative pathogen that has emerged as one of the most highly antibiotic-resistant bacteria worldwide. Multidrug efflux within these highly drug-resistant strains and other opportunistic pathogens is a major cause of failure of drug-based treatments of infectious diseases. The best-characterized multidrug efflux system in A. baumannii is the prevalent Acinetobacter drug efflux B (AdeB) pump, which is a member of the resistance-nodulation-cell division (RND) superfamily. Here, we report six structures of the trimeric AdeB multidrug efflux pump in the presence of ethidium bromide using single-particle cryoelectron microscopy (cryo-EM). These structures allow us to directly observe various novel conformational states of the AdeB trimer, including the transmembrane region of trimeric AdeB can be associated with form a trimer assembly or dissociated into “dimer plus monomer” and “monomer plus monomer plus monomer” configurations. We also discover that a single AdeB protomer can simultaneously anchor a number of ethidium ligands and that different AdeB protomers can bind ethidium molecules simultaneously. Combined with molecular dynamics (MD) simulations, we reveal a drug transport mechanism that involves multiple multidrug-binding sites and various transient states of the AdeB membrane protein. Our data suggest that each AdeB protomer within the trimer binds and exports drugs independently.

## INTRODUCTION

Acinetobacter baumannii is an opportunistic Gram-negative pathogen that has emerged as one of the most problematic and highly antibiotic-resistant bacteria in the world. This bacterial pathogen exhibits a high level of antimicrobial resistance (AMR) to a broad spectrum of agents (1, 2). To date, emerging carbapenem-resistant A. baumannii is listed in the highest AMR threat category by the CDC, with carbapenem resistance now found in the majority of A. baumannii strains worldwide (3, 4). Correspondingly, the WHO also lists carbapenem-resistant A. baumannii as a first-priority pathogen for the research and development of new antibiotics (5). Unfortunately, there is an increasing trend of resistance to even the last-resort antibiotics, such as colistin and tigecycline (6–9). These resistant strains are essentially nonsusceptible to every FDA-approved antibiotic and, hence, are untreatable. Currently, there is no standard treatment regimen for Acinetobacter infections, and empirical therapy is not always reliable. In addition, A. baumannii can survive for prolonged periods in clinical settings, potentiating its spread as a nosocomial pathogen affecting our most vulnerable patients (10, 11).

Multidrug efflux is considered one of the major causes of the failure of drug-based treatments of infectious diseases (12). In A. baumannii, the best-characterized multidrug efflux system is the prevalent Acinetobacter drug efflux ABC (AdeABC) tripartite system (13). This efflux system is capable of mediating resistance to a broad spectrum of clinically relevant antimicrobial agents, such as aminoglycosides, tetracyclines, macrolides, β-lactams, fluoroquinolones, chloramphenicol, and trimethoprim (1, 13–20). The AdeABC locus consists of three tandemly linked genes encoding the AdeA periplasmic membrane fusion protein, the AdeB multidrug efflux pump, which is an inner membrane protein belonging to the hydrophobe-amphiphile efflux resistance-nodulation-cell division (HAE-RND) family of efflux transporters, and the AdeC outer membrane channel protein (13, 21). It should be noted that not all A. baumannii strains carry the *adeC* gene. However, it has been demonstrated that the presence of *adeC* can elevate the levels of resistance to several antibiotics (22).

To understand the mechanisms of substrate recognition and extrusion of the A. baumannii AdeB multidrug efflux pump, here, we define cryoelectron microscopy (cryo-EM) structures of this membrane protein embedded in lipidic nanodiscs in the presence of ethidium bromide (Et). The approach of using lipidic nanodiscs is beneficial to structural determination of this membrane protein, as we can avoid the harsh detergent environment during the process of single-particle imaging. In addition, cryo-EM has the ability to study various conformational states of biomacromolecules within a single sample (23, 24), accelerating the elucidation of the conformations of various transient states that the multidrug efflux pump must go through during the drug transport cycle. Recently, we developed a novel iterative methodology, termed “build and retrieve” (BaR), to process heterogeneous sample data sets for high-resolution structural determination (25). It is a powerful technique that enables us to sort and separate cryo-EM images into various proteins and/or different conformational classes of the same protein. With the cryo-EM approach, we are able to observe detailed structural information of various transient states that are crucial for understanding the mechanisms of multidrug recognition and extrusion of the AdeB pump. Here, we present six cryo-EM structures of the trimeric A. baumannii AdeB multidrug efflux pump either alone or bound with Et molecules. Interestingly, these structural data allow us to directly observe two new phenomena, that a single AdeB protomer can simultaneously accommodate multiple Et ligands and that different AdeB protomers within the trimer are capable of binding Et simultaneously. Additionally, we notice that the transmembrane region of trimeric AdeB can switch its oligomerization from “trimer” to “dimer plus monomer” and “monomer plus monomer plus monomer” configurations. We also investigate the functional dynamics of this transporter using molecular dynamics (MD) and target MD (TMD) simulations. Combined, our data provide a detailed pathway for substrate transport via the AdeB membrane protein.

## RESULTS

### Structural determination of the AdeB multidrug efflux pump.

To confirm that substrates bind A. baumannii AdeB with sufficient affinity to visualize via structural analysis, we first purified the full-length multidrug efflux pump and used the technique of fluorescence polarization to quantify the interactions of purified AdeB with ethidium bromide (Et) and rhodamine 6G (R6G). The titration experiments indicate that AdeB specifically binds these two substrates in the micromolar range. The measured dissociation constant (*K_D_*) values for Et and R6G are 2.5 ± 0.1 μM and 3.1 ± 0.1 μM, respectively (see Fig. S1 in the supplemental material). These *K_D_*s are similar to those measured for the Escherichia coli AcrB multidrug efflux pumps (26) using the same technique.

10.1128/mBio.03690-20.1 FIG S1 AdeB and ethidium interactions. (A) Fluorescence polarization (FP) assay shows Et binds to AdeB with an affinity of 2.5 ± 0.1 μM. (B) FP shows R6G binds AdeB with an affinity of 3.1 ± 0.1 μM. (C) Kinetics of intracellular accumulation of Et in the A. baumannii AbΔ3 cells lacking the AdeABC, AdeIJK, and AdeFGH efflux pumps. Et accumulates inside cells with all three pump systems knocked out (green, 0 μM Et; blue, 0.5 μM Et; yellow, 1 μM Et; gray, 2 μM Et; red, 4 μM Et; and cyan, 8 μM Et). (D) Complementing triple knockout cells with AdeABC restores extrusion and halts accumulation of Et inside cells (green, 0 μM Et; blue, 0.5 μM Et; yellow, 1 μM Et; gray, 2 μM Et; red, 4 μM Et; and cyan, 8 μM Et). Download FIG S1, PDF file, 0.4 MB. Copyright © 2021 Morgan et al. 2021 Morgan et al. https://creativecommons.org/licenses/by/4.0/ This content is distributed under the terms of the Creative Commons Attribution 4.0 International license.

A. baumannii encodes seven RND-type efflux pumps (17), with AdeABC and AdeIJK being responsible for resistance to most clinically relevant antibiotics. However, the expression level of AdeABC is the highest among these tripartite efflux pumps (27). We used an A. baumannii *ΔadeABC ΔadeFGH ΔadeIJK* (AbΔ3) triple-knockout strain (28), which harbors deletions in the chromosomal *adeABC*, *adeFGH*, and *adeIJK* genes, from the clinical MDR isolate AYE. We found that the MICs of Et in the efflux-deficient AbΔ3 cells were 4 to 8 mg/liter, but the MIC was increased to 32 to 64 mg/liter when these AbΔ3 cells were complemented with the plasmid-borne AdeABC pump. Further, Et readily accumulated in efflux-deficient hyperporinated AbΔ3-Pore cells (28), as seen from the increase in Et fluorescence of these cells in concentration- and time-dependent manners (Fig. S1). In contrast, no significant time-dependent accumulation of Et was observed in the AdeABC-overproducing AbΔ3-Pore(pAdeABC) cells (28) (Fig. S1). Combined with fluorescence polarization, these data strongly indicate that Et is a substantive substrate of the AdeB multidrug efflux pump.

We then reconstituted the purified AdeB membrane protein into lipidic nanodiscs. We incubated 2 μM AdeB-nanodisc sample with 20 μM Et for 2 h and collected single-particle cryo-electron microscopy (cryo-EM) images of the AdeB-Et complex. Cryo-EM detects single-particle images at random orientations in a solution state where the protein molecules are free to change conformation to accommodate substrate binding and transport. Therefore, we believe that these cryo-EM images should contain invaluable information regarding different intermediates the pump must adopt within the transport cycle. Extensive classification of the single-particle images indicated that there were six distinct populations of AdeB with different conformations coexisting in the single nanodisc sample (Fig. S2). Several iterative rounds of classifications allowed us to sort the images based on these six distinct conformations. Surprisingly, three of these structures depicted that the trimeric AdeB pump does not contain any bound ligands. For the other three AdeB structures, it was observed that one of the AdeB protomers within the trimer possesses two or three bound Et molecules. Interestingly, one of these three AdeB-Et complex structures showed that two independent AdeB protomers within the trimer can simultaneously anchor Et ligands.

10.1128/mBio.03690-20.2 FIG S2 AdeB processing workflow. (A) Processing of 5,665 micrographs to get initial pool of 982,491 particles. (B) 3D classification results. (C) Two 3D classes gave rise to consensus refinement with 682,177 total particles. (D) Focused classification of only the periplasmic domains using 3D variability analysis (3DVA) gave rise to two different populations, the apo form (protomers closed) and the bound form (one or two protomers open). (E) Further classification and cleaning by 3DVA gave rise to six total structures (3 apo and 3 bound) with marked differences. (F) Final density-modified maps and resolutions. Download FIG S2, PDF file, 0.1 MB. Copyright © 2021 Morgan et al. 2021 Morgan et al. https://creativecommons.org/licenses/by/4.0/ This content is distributed under the terms of the Creative Commons Attribution 4.0 International license.

### Structures of apo-AdeB.

**FIG 1:**
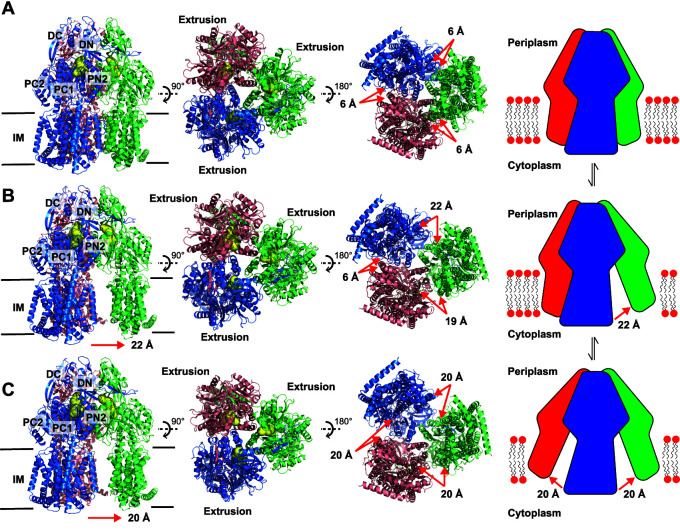
Cryo-EM structures of AdeB-I, AdeB-II, and AdeB-III. (A) Ribbon diagram of the structure of the side view (viewed in the membrane plane), top view (viewed from the extracellular space), and bottom view (viewed from the cytoplasm) of the AdeB-I trimer. A cartoon indicating the trimer configuration at the transmembrane domain is included. (B) Ribbon diagram of the structure of the side view, top view, and bottom view of the AdeB-II trimer. A cartoon indicating the dimer plus monomer configuration at the transmembrane domain is included. (C) Ribbon diagram of the structure of the side view, top view, and bottom view of the AdeB-III trimer. A cartoon indicating the monomer plus monomer plus monomer configuration at the transmembrane domain is included. In panels A to C, the AdeB extrusion protomers are colored blue, green, and pink, respectively. Each protomer creates an extrusion channel (colored yellow) for drug export. The switch in configuration of the trimeric AdeB pump from trimer (A) to dimer plus monomer (B) and monomer plus monomer plus monomer (C) may alter the interactions between the AdeB pump and AdeA adaptor at the protein-protein interface.

Three distinct conformations of apo-AdeB (AdeB-I, AdeB-II, and AdeB-III) were captured in our cryo-EM data (Fig. 1, Fig. S2 and S3, and Table S1). All of these structures depict that AdeB adopts the fold of a typical HAE-RND-type protein and forms a homotrimer, with its 3-fold symmetrical axis positioned perpendicular to the membrane surface. Each subunit of AdeB contains 12 transmembrane helices (TM1 to TM12) and six periplasmic subdomains (PN1, PN2, PC1, PC2, DN, and DC). The assignments of the AdeB protomers are shown in Fig. S4 and Table S2.

10.1128/mBio.03690-20.3 FIG S3 Cryo-EM analysis of apo AdeB. (A) AdeB-I cryo-EM results. (B) AdeB-II cryo-EM results. (C) AdeB-III cryo-EM results. Panels depict representative 2D classes (top left), GS-FSC resolution curve (top right), final cryo-EM map (bottom left), and representative density (bottom right) for each structure. Amino acids are represented as cyan sticks. Download FIG S3, PDF file, 0.2 MB. Copyright © 2021 Morgan et al. 2021 Morgan et al. https://creativecommons.org/licenses/by/4.0/ This content is distributed under the terms of the Creative Commons Attribution 4.0 International license.

10.1128/mBio.03690-20.4 FIG S4 AdeB protomer classification. (A) Example extrusion tunnel measurements for resting (top left), access* (top right), binding (bottom left), and extrusion (bottom right) states. In the resting (AdeB-Et-II chain A), access* (AdeB-Et-III chain A), and binding (AdeB-Et-I chain C) states, the extrusion tunnel is closed, with distances of 9.19 Å, 9.38 Å, and 9.40 Å, respectively, between the Cα atoms of Q125 and Y749. In the extrusion protomer (AdeB-Et-I chain B), the extrusion tunnel is open with a measured distance of 14.01 Å between Q125 and Y749, allowing export of ligand. (B) Proton relay network measurements. In the resting protomer (AdeB-Et-II chain A, top left), the NZ atom of K931 is within hydrogen bonding distance of the O atoms of N932 and T968. In the access* protomer (AdeB-Et-III chain A, top right), K931 swings away from N932 and T968 to interact with D407. In the binding protomer (AdeB-Et-I chain C, bottom left), NZ of K931 interacts with the O atoms of D407 and D408. In the extrusion protomer (AdeB-II chain B, bottom right), K931 swings back to interact with N932 and T968, where NZ of K931 is within hydrogen bonding distance of O atoms on N932 and T968. Download FIG S4, PDF file, 0.9 MB. Copyright © 2021 Morgan et al. 2021 Morgan et al. https://creativecommons.org/licenses/by/4.0/ This content is distributed under the terms of the Creative Commons Attribution 4.0 International license.

10.1128/mBio.03690-20.9 TABLE S1 Cryo-EM data collection and refinement statistics. Download Table S1, PDF file, 0.03 MB. Copyright © 2021 Morgan et al. 2021 Morgan et al. https://creativecommons.org/licenses/by/4.0/ This content is distributed under the terms of the Creative Commons Attribution 4.0 International license.

10.1128/mBio.03690-20.10 TABLE S2 Different transient states of the AdeB protomers. Download Table S2, PDF file, 0.04 MB. Copyright © 2021 Morgan et al. 2021 Morgan et al. https://creativecommons.org/licenses/by/4.0/ This content is distributed under the terms of the Creative Commons Attribution 4.0 International license.

### (i) Structure of AdeB-I.

In the structure of AdeB-I, the three AdeB protomers are identical in conformation, displaying the “extrusion” state of the pump (Fig. 1, Fig. S3, and Table S1). The periplasmic cleft created by subdomains PC1 and PC2 is closed. An extrusion channel is found in each AdeB protomer (Fig. 1). It is interesting that the three AdeB molecules tightly interact with each other, both in the transmembrane and the periplasmic regions, to form a trimer in this state. Within the transmembrane domain, six phosphatidylethanolamine (PE) molecules were observed to bind at the outer leaflet region of the trimeric AdeB pump (Fig. S5). Each AdeB protomer forms two distinct PE lipid-binding sites. One of these two binding sites is located at the interior surface of the large central cavity of the trimeric AdeB efflux pump, with each individual AdeB protomer solely responsible for its binding. The other lipid binding site is found within the protomer-protomer interface, where two AdeB protomers interact with each other within their transmembrane regions to anchor this bound PE molecule. It appears that the TM-associated PE molecule may help promote these protomer-protomer interactions within the subunit interface.

10.1128/mBio.03690-20.5 FIG S5 Lipid binding sites. (A) AdeB-I has six bound lipids in the transmembrane domain, one between each protomer and another on each individual protomer. Colored boxes correspond to a blown-up view. (B) AdeB-II has three bound lipids in the transmembrane domain, one between the purple and pink protomers, one on the purple protomer, and another on the pink protomer. It appears that the green protomer, which does not interact with the purple and pink protomer at the transmembrane region, does not have any bound lipids. (C) No lipids are present at the transmembrane domain of AdeB-III. The three protomers are 20 Å away from each other, as measured between V10 and A881 Cα atoms. Download FIG S5, PDF file, 0.2 MB. Copyright © 2021 Morgan et al. 2021 Morgan et al. https://creativecommons.org/licenses/by/4.0/ This content is distributed under the terms of the Creative Commons Attribution 4.0 International license.

### (ii) Structure of AdeB-II.

Like AdeB-I, the three AdeB protomers of the second apo-AdeB structure, designated AdeB-II, present an extrusion conformation, where the three periplasmic clefts of the AdeB trimer are closed and a channel for extrusion is found in each protomer (Fig. 1, Fig. S3, and Table S1). However, in the AdeB-II structure, it is observed that the organization of the AdeB trimer is packed in a way that can be interpreted as a dimer plus monomer configuration within the transmembrane domain (Fig. 1). The region that maintains the assembly of the pump in the trimeric oligomerization state is the periplasmic domain, where the long-arm feature formed by subdomain DN strongly coordinates with the hook-like structural feature of subdomain DC of the neighboring AdeB subunit to secure intersubunit interactions within the trimer. This region also contributes to form a hinge so that the transmembrane domain of each protomer is capable of shifting toward and away from each other. Within the two protomers interacting with each other at the transmembrane region, we observed three bound PE lipids. One of these lipids is located at the interface between the two AdeB protomers. The other two lipid molecules are found to bind by individual protomers (Fig. S5). No lipid is observed to associate with the third AdeB protomer.

### (iii) Structure of AdeB-III.

Strikingly, for the AdeB-III structure, the three AdeB protomers largely organize in a monomer plus monomer plus monomer configuration in the transmembrane region (Fig. 1, Fig. S3, and Table S1). In this conformational state, the three transmembrane domains of the AdeB trimer are significantly distant from each other and do not seem to participate in intersubunit interactions, although each AdeB protomer still presents an extrusion conformation of the pump with an extrusion channel creating at the periplasmic domain (Fig. 1). Interestingly, no noticeable lipid ligand was bound by these three AdeB protomers (Fig. S5). Like the dimer plus monomer configuration of the AdeB-II structure, the three AdeB protomers of AdeB-III specifically contact each other via the periplasmic domains, where DN of one AdeB subunit interacts with DC of another subunit to maintain trimeric oligomerization of the pump.

Previously, a cryo-EM structure of the AcrAB-TolC (29) and X-ray structure of the CusAB (30) efflux complexes indicated that the adaptor proteins AcrA and CusB specifically interact with the efflux pumps AcrB and CusA, respectively. In particular, the 2.9-Å-resolution structure of CusAB depicted that domains 1 and 2 (corresponding to the membrane-proximal and β-barrel domains) of the CusB adaptor distinctly contacts the periplasmic subdomains PN2, PC1, and PC2 of the CusA pump via charge-charge, charge-dipole, and dipole-dipole interactions (30). Isothermal titration calorimetry further suggested that the binding affinity between CusA and CusB is 5.1 μM with stoichiometry of a 1:2 CusA-to-CusB molar ratio (30). We suspect that the switch in configuration of the trimeric AdeB pump from trimer to dimer plus monomer and monomer plus monomer plus monomer can alter the interactions between the AdeB pump and AdeA adaptor at the protein-protein interface, in turn facilitating the transport of substrates across the periplasmic space of A. baumannii.

### Structures of AdeB-Et.

In addition to the three apo-AdeB structures, we also observed three distinct structures of AdeB bound with Et in our cryo-EM sample. These three different AdeB-Et complex structures were labeled as AdeB-Et-I, AdeB-Et-II, and AdeB-Et-III (Fig. S2, Fig. S6, and Table S1). Interestingly, the cryo-EM data indicate that all of these Et-bound structures present asymmetric trimers, where two or three protomers within each trimer are unique and display different conformational states.

10.1128/mBio.03690-20.6 FIG S6 Cryo-EM analysis of AdeB-Et. (A) AdeB-Et-I cryo-EM results. (B) AdeB-Et-II cryo-EM results. (C) AdeB-Et-III cryo-EM results. Panels depict representative 2D classes (top left), GS-FSC resolution curve (top right), final cryo-EM map (bottom left), and representative density (bottom right) for each structure. Amino acids are represented as cyan sticks. Download FIG S6, PDF file, 0.2 MB. Copyright © 2021 Morgan et al. 2021 Morgan et al. https://creativecommons.org/licenses/by/4.0/ This content is distributed under the terms of the Creative Commons Attribution 4.0 International license.

### (i) Structure of AdeB-Et-I.

**FIG 2:**
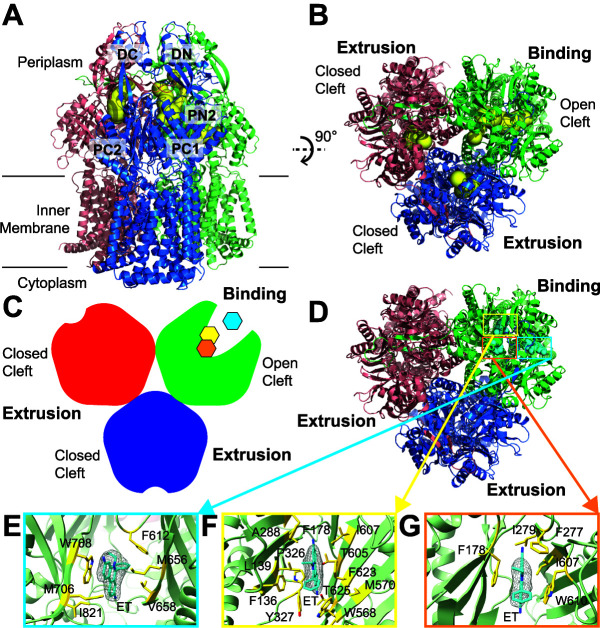
Cryo-EM structure of AdeB-Et-I. (A) Ribbon diagram of the structure of AdeB-Et-I viewed in the membrane plane. (B) Ribbon diagram of the structure of AdeB-Et-I viewed from the extracellular space (top view). In panels A and B, the one binding and two extrusion protomers of AdeB are colored green, pink, and blue, respectively. The binding and extrusion channels are colored yellow. (C) A cartoon representing the structure of the AdeB trimer viewed from the extracellular space. The one binding and two extrusion protomers of AdeB are colored green, red, and blue, respectively. The three bound Et ligands in the binding protomer are colored cyan (at the entrance binding site), yellow (at the distal binding site), and orange (at the hydrophobic patch binding site). (D) Top view of the AdeB trimer depicting three bound Et ligands located at the binding protomer. The one binding and two extrusion protomers of AdeB are colored green, pink, and blue, respectively. The three bound Et ligands are depicted as cyan spheres. (E to G) The Et binding sites. Shown is an enlarged view of the entrance (E), distal (F), and hydrophobic patch (G) sites. The EM densities of bound Et ligands are shown as gray meshes. The bound Et ligands are represented as cyan sticks. Residues that are involved in Et binding are represented as yellow sticks. The secondary structural elements of the binding protomer are depicted as green ribbons.

Surprisingly, the structure of AdeB-Et-I depicts that one of the AdeB protomers prefers a transient state with its periplasmic cleft open, whereas the periplasmic clefts of the other two protomers remain closed. These two protomers with the periplasmic cleft closed represent the extrusion conformation, as each protomer possesses an extrusion channel at the periplasmic domain (Fig. 2, Fig. S6, and Table S1). The conformation of the AdeB protomer with the open cleft presents the “binding” state of the pump. Within the binding protomer, an elongated channel allows for the interior of the periplasmic domain to be exposed to solvent. Unexpectedly, three extra densities were found in three distinct locations inside this open cleft. The shape of each density is compatible with Et, indicating that three Et molecules are simultaneously bound by this binding protomer. The three bound Et molecules appear to line the elongated channel formed by the protomer, depicting a path for drug transport. The first bound Et is situated at the entrance of the periplasmic cleft, where the entrance residues M656, V658, M706, W708, and I821 are responsible for providing hydrophobic and aromatic interactions to anchor-bound Et (Fig. 2). It should be noted that the corresponding AdeB residues M656, V658, and W708 are F664, F666, and R717 in the AcrB pump. These AcrB residues have been shown to be important for drug recognition (31–33). Interestingly, the gate loop (G-loop) is observed to swing toward the periplasmic entrance in this conformational state, enabling the G-loop residue F612 to come closer to bound Et (within 4.5 Å), forming an aromatic interaction to further stabilize Et binding. In AcrB, the G-loop phenylalanine is F617. Molecular dynamics simulations suggested that this AcrB F617 residue is important for substrate binding and extrusion (32).

The second Et molecule is bound at the same horizontal level compared with that of the first bound Et. This bound Et is located at the distal drug-binding site approximately 20 Å above the membrane surface. Within 4.5 Å of this bound Et, residues F136, L139, F178, A288, P326, Y327, W568, M570, T605, I607, F623, and T625 are responsible for securing the binding of this bound Et (Fig. 2). The AdeB F178 residue corresponds to F178 in AcrB. This AcrB residue has been found to be involved in minocycline binding (34).

The third bound Et is observed to line up right above the second bound Et with respect to the membrane surface. Of note, the third Et is observed to entangle in the hydrophobic patch of the distal binding site. We designated this site a hydrophobic patch drug-binding site. Residues F178, F277, I279, I607, and W610 are engaged in anchoring this Et (Fig. 2). The corresponding hydrophobic patch residues in AcrB, including the AcrB residues F178, I277, V612, and F615, have been found to have the greatest impact on drug binding (32). Interestingly, the second bound Et in the distal drug-binding site is also within 4.5 Å and performs an aromatic interaction with the third bound Et at the hydrophobic patch site to secure the binding.

### (ii) Structure of AdeB-Et-II.

**FIG 3:**
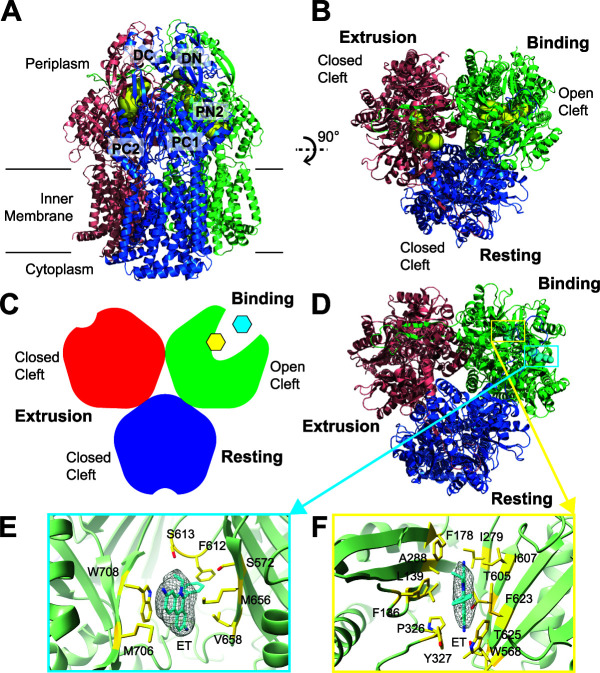
Cryo-EM structure of AdeB-Et-II. (A) Ribbon diagram of the structure of AdeB-Et-II viewed in the membrane plane. (B) Ribbon diagram of the structure of AdeB-Et-II viewed from the extracellular space (top view). In panels A and B, the binding, extrusion, and resting protomers of AdeB are colored green, pink, and blue, respectively. The binding and extrusion channels are colored yellow. No channel is observed in the periplasmic domain of the resting protomer. (C) A cartoon representing the structure of the AdeB trimer viewed from the extracellular space. The binding, extrusion, and resting protomers of AdeB are colored green, red, and blue, respectively. The two bound Et ligands in the binding protomer are colored cyan (at the entrance binding site) and yellow (at the distal binding site). (D) Top view of the AdeB trimer depicting two bound Et ligands located at the binding protomer. The binding, extrusion, and resting protomers of AdeB are colored green, pink, and blue, respectively. The two bound Et ligands are depicted as cyan spheres. (E and F) The Et binding sites. Shown is an enlarged view of the entrance (E) and distal (F) sites. The EM densities of bound Et ligands are shown as gray meshes. The bound Et ligands are represented as cyan sticks. Residues that are involved in Et binding are represented as yellow sticks. The secondary structural elements of the binding protomer are depicted as green ribbons.

Similar to AdeB-Et-I, the structure of AdeB-Et-II indicates that the periplasmic cleft of one of the protomers is open and the clefts of the other two protomers are in closed conformation. A detailed inspection reveals that the conformational states of these three AdeB protomers within the AdeB-Et-II complex resemble the crystal structure of the Campylobacter jejuni CmeB multidrug efflux pump (35), where the three CmeB protomers present the resting, extrusion, and binding conformations (Fig. 3, Fig. S6, and Table S1). Like the CmeB structure, no channel was found in the AdeB resting conformer. As expected, an extrusion channel was observed in the AdeB extrusion protomer, and an elongated channel that leads through the opening of the periplasmic cleft was identified in the AdeB binding protomer (Fig. 3, Fig. S6, and Table S1).

Within the binding protomer of AdeB, the cryo-EM images depict two extra densities corresponding to two bound Et molecules that were found at the entrance and distal drug-binding sites, respectively. However, there were no noticeable extra densities in the drug-binding sites of the resting and extrusion protomers. The first bound Et is anchored in a way very similar to that found in the entrance drug-binding site of AdeB-Et-I. The entrance binding site residues S572, M656, V658, M706, W708, and N709 of AdeB-Et-II attach this Et at the periplasmic entrance of AdeB-Et-II. The G-loop residues F612 and S613 also contribute to this binding (Fig. 3). The second bound Et is found within the distal drug-binding site of AdeB-Et-II, where F136, L139, F178, I279, A288, P326, Y327, W568, T605, I607, T625, and F623 are responsible for the binding (Fig. 3). Interestingly, both Et molecules are bound approximately 2 Å above that of their corresponding Et found in AdeB-Et-I with respect to the membrane surface.

### (iii) Structure of AdeB-Et-III.

**FIG 4:**
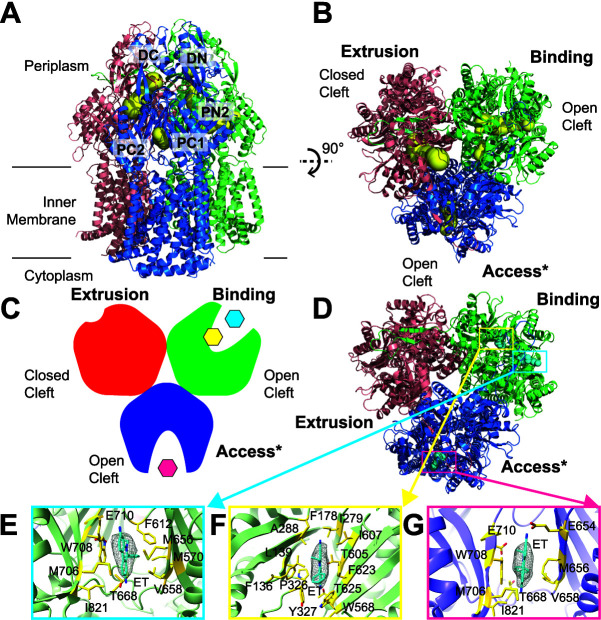
Cryo-EM structure of AdeB-Et-III. (A) Ribbon diagram of the structure of AdeB-Et-III viewed in the membrane plane. (B) Ribbon diagram of the structure of AdeB-Et-III viewed from the extracellular space (top view). In panels A and B, the binding, extrusion, and access* protomers of AdeB are colored green, pink, and blue, respectively. The extrusion, binding and access* channels are colored yellow. (C) A cartoon representing the structure of the AdeB trimer viewed from the extracellular space. The binding, extrusion, and access* protomers of AdeB are colored green, red, and blue, respectively. The two bound Et ligands in the binding protomer are colored cyan (at the entrance binding site) and yellow (at the distal binding site). The bound Et ligand in the access* protomer is colored magenta (at the entrance binding site). (D) Top view of the AdeB trimer depicting two bound Et ligands at the binding protomer and one Et ligand at the access* protomer. The binding, extrusion, and access* protomers of AdeB are colored green, pink, and blue, respectively. The three bound Et ligands in the binding and access* protomers are represented as cyan spheres. (E to G) The Et binding sites of the binding and access* protomers. Shown is an enlarged view of the entrance site of the binding protomer (E), distal site of the binding protomer (F), and entrance site of the access* protomer (G). In panels E to G, the EM densities of bound Et ligands are show as gray meshes. The bound Et ligands are represented as cyan sticks. Residues that are involved in Et binding are represented as yellow sticks. The secondary structural elements of the binding and access* protomers are depicted as green and purple ribbons, respectively.

The trimeric AdeB-Et-III structure also exhibits an asymmetric conformation, with three distinct conformers in the trimer (Fig. 4, Fig. S6, and Table S1). It is clear that one AdeB protomer presents the binding state and a second AdeB protomer depicts the extrusion form of the pump. The third AdeB protomer displays a conformation that is more or less in good agreement with the “access” conformation of AcrB (34) and MtrD (36). Unexpectedly, a bound Et ligand was found within the entrance drug-binding site of this protomer. Therefore, we labeled this conformer the “access*” form of AdeB. The structure of AdeB-Et-III is distinct from all known structures of the RND-type efflux pumps in that two independent AdeB protomers within the trimer are found to be occupied by substrates. This structural information strongly indicates that each AdeB protomer within the trimer can independently bind and export substrates. All of these different conformations most likely represent various intermediates that the AdeB pump must go through during the transport cycle to extrude drugs from bacterial cells.

In the binding conformer of AdeB-Et-III, there are two Et molecules occupying the periplasmic cleft. One Et molecule is found to bind at the entrance site, where residues F612, M656, V658, T668, W708, E710, and I821 are responsible for anchoring this ligand (Fig. 4). The second Et molecule is observed to bind at the deep distal-binding pocket, with F136, L139, F178, I279, A288, P326, Y327, W568, T605, I607, F623, and T625 participating in securing Et binding (Fig. 4). Within the access* conformer of AdeB-Et-III, an Et ligand is seen to occupy the entrance drug-binding site. Residues Q575, E654, M656, V658, M706, W708, E710, and I821 are engaged in the binding (Fig. 4). Like the structures of AdeB-Et-I and AdeB-Et-II, no Et ligand is found to bind in the extrusion protomer of AdeB.

### Computational simulation of the AdeB multidrug efflux pump.

To understand the molecular mechanism of drug transport via the AdeB pump, we performed molecular dynamics simulations based on the cryo-EM structures of the AdeB pump both in the absence (an extrusion protomer of the AdeB-Et-I structure) and presence (the binding protomer of the AdeB-Et-I structure) of Et ligands. One-microsecond production MD simulations were conducted for each protomer (Fig. S7). It appears that the conformations of the flexible loop (F-loop; residues 661 to 670), which connects the entrance and proximal sites of the periplasmic cleft, and the G-loop (residues 609 to 618) are directly related to the opening and closing of the periplasmic cleft. In the extrusion state, the periplasmic cleft is closed. It is observed that both the F- and G-loops contribute to block the periplasmic cleft and occlude the entrance and the proximal sites. In contrast, in the binding state, the F- and G-loops are positioned so that these binding sites open up and are made available for the ligands. We measured the minimum distances between residues F612 of the G-loop and T668 of the F-loop as a function of time in two different MD simulations, utilizing the structures of the extrusion protomer of AdeB-I and binding protomer of AdeB-Et-I. The simulations indicate that the minimum distances between these two residues are between 3 and 4 Å for the extrusion protomer, whereas these distances are between 4 and 15 Å in the binding protomer (Fig. S7). It appears that the conformation of the extrusion state is stabilized by hydrophobic interactions between residues T668, F669, and F612.

10.1128/mBio.03690-20.7 FIG S7 Results of 1-μs simulation. (A) MD simulation results of the AdeB-Et-I extrusion protomer (black) and AdeB-Et-I binding protomer (red). The Cα atom RMSD (root mean square deviations) are based on the MD simulation trajectories (1 μs). (B) Distances between residues F612 and T668 over the course of the simulation (1 μs) for both extrusion (black) and binding (red) protomers from AdeB-Et-I. (C) The G-loop (cyan) and F-loop (yellow) form a gate that is closed in the extrusion protomer (blue cartoon). Residues F612 (cyan spheres) and T668 (yellow spheres) interact through hydrophobic interactions to close the gate. In the binding protomer (green cartoon), the F- and G-loops separate to allow ligand entry. (D) RMSD comparison of F612 and nearby residue F569 over the 1-μs simulation. (E) RMSD comparison of W610 and nearby residue W568. W610 and F612 are critical for the transport of ligand out of the pump. Download FIG S7, PDF file, 1.5 MB. Copyright © 2021 Morgan et al. 2021 Morgan et al. https://creativecommons.org/licenses/by/4.0/ This content is distributed under the terms of the Creative Commons Attribution 4.0 International license.

We then calculated the root mean square deviation (RMSD) of residues F612 and W610, located at the G-loop, as a function of time for the binding protomer. We observed that these two residues have very large RMSD values compared with the nearby residues F569 and W568, suggesting that F612 and W610 are highly flexible and very dynamic in nature (Fig. S7). It appears that the aromatic sidechains of these residues act as a “broom” to shuttle the bound ligand molecule out of the pump. This observation is indeed in good agreement with molecular dynamics simulations that suggest the AcrB F617 residue (corresponding to F612 of AdeB) is important for drug transport (32).

**FIG 5:**
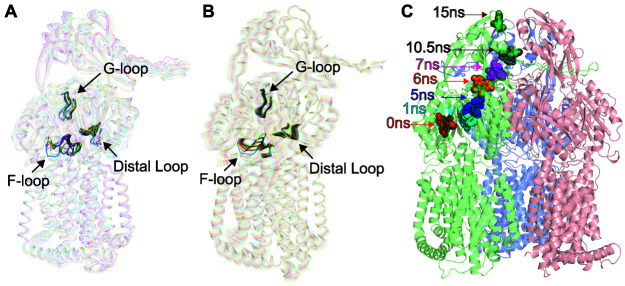
MD and target MD simulations of the AdeB efflux pump. (A) Superimposition of the cryo-EM structures of 18 protomers of AdeB (cryo-EM structures of six AdeB trimers, and each trimer possesses three protomers). The structures indicate that the most obvious differences of these structures represent the change in conformations of the F-loop, G-loop, and another flexible loop (residues 131 to 139, designated the distal loop), in addition to the relative movements of secondary structural elements making up the left side (residues 693 to 709 and 819 to 833 of PC2) and right side (residues 624 to 650 of PC1) of the periplasmic cleft. (B) MD simulations of the AdeB efflux pump. Consistent with the 18 structures of the AdeB protomers, the first eigenvector based on PCA suggests that the major motions of AdeB are localized at the F-loop, G-loop, and distal loop, in addition to the relative movements of secondary structural elements making up cleft between PC1 and PC2. (C) Target MD simulations of the AdeB pump. The calculations depict snapshots (0, 1, 5, 6, 7, 10.5, and 15 ns) of Et shuttling within the periplasmic domain of AdeB.

Interestingly, principal component analysis (PCA) based on the AdeB binding and extrusion protomers of the AdeB-Et-I structure indicates that the first eigenvector, which depicts the most important motion extracted from the MD simulation trajectory, represents the motions of the F-loop, G-loop, and another flexible loop (residues 131 to 139), all part of the distal drug-binding site, in addition to the relative movements of secondary structural elements making up the left side (residues 693 to 709 and 819 to 833 of PC2) and right side (residues 624 to 650 of PC1) of the periplasmic cleft. This result is in good agreement with our cryo-EM structures, depicting that the major conformational changes are the flexible loops inside the periplasmic cleft beside the opening and closing of the cleft between subdomains PC1 and PC2 (Fig. 5). These data strongly suggest that there are several coordinated motions within the entrance, proximal, and distal drug-binding sites to help shuttle the bound drug molecule traveling through the channel formed by the AdeB protomer for drug export.

To further elucidate the mechanism of substrate transport, we performed target MD simulations using the NAMD program. We observed that the Et ligand indeed follows the path of the binding and extrusion channels to shuttle along the AdeB pump (Fig. 5). Based on the target MD calculations, Et first enters the AdeB pump via the entrance binding site, which includes residues M656 and W706. It then passes through the G-loop, reaches the hydrophobic patch binding site, and eventually leaves this membrane protein through the exit site formed by Q125 and Y749 (Fig. S8).

10.1128/mBio.03690-20.8 FIG S8 Targeted MD simulation. (A) Highlighted time points for focus on interactions between Et and AdeB (0 ns and 7 ns). Box colors correspond to blown-up images. (B) Focused images of AdeB-Et interactions at selected time points from TMD simulation. Locations correspond to the entrance binding site (red) and ethidium leaving through the extrusion tunnel (magenta). Et is colored cyan, while all residues within 4.5 Å are highlighted with yellow sticks in all images. Download FIG S8, PDF file, 1.3 MB. Copyright © 2021 Morgan et al. 2021 Morgan et al. https://creativecommons.org/licenses/by/4.0/ This content is distributed under the terms of the Creative Commons Attribution 4.0 International license.

## DISCUSSION

We have defined cryo-EM structures of the AdeB multidrug efflux pump in the presence of Et. These structural data allow us to observe new conformational states that have not been identified before for the RND efflux pumps. It appears that the transmembrane region of trimeric AdeB can be associated to form a trimer assembly or dissociated into dimer plus monomer and monomer plus monomer plus monomer configurations. The motion that governs the association and dissociation can be interpreted as a simple rigid-body rotation that allows the transmembrane regions of the AdeB protomers to tilt away from each other. The hinge of this long-distance conformational change is located at the periplasmic docking domain of the AdeB trimer, where the intersubdomains DN and DC are responsible for tethering the trimeric assembly of this pump. We postulate that this rigid-body rotational motion can trigger a conformational change of the AdeA membrane fusion protein. Similar to the assembly characteristics of the membrane fusion proteins CusA and AcrA seen in the CusAB (30) and AcrAB-TolC (29) complexes, AdeA is presumed to assemble as a hexameric channel to interact with the trimeric AdeB pump. The rigid-body rotational motion of AdeB may be responsible for controlling the opening and closing of the AdeA hexameric channel to facilitate drug extrusion. Thus far, there is no known evidence that AdeB can interact with membrane fusion proteins other than AdeA to function. However, it has been documented that the Pseudomonas aeruginosa MexB multidrug efflux pump can form a functional complex with the E. coli AcrA membrane fusion protein (37). The multiple states of AdeB observed in the cryo-EM structures of AdeB may facilitate the interaction between AdeB and other membrane fusion proteins. The possibility that AdeB can associate with other membrane fusion proteins awaits further investigation.

Surprisingly, we only observed the trimer configuration at the transmembrane domain of the Et-bound AdeB structures. This indicates that the presence of substrate stabilizes the trimer assembly. One interpretation is that the conformation of the trimer state may be more compatible with AdeA binding, which in turn facilitates the extrusion of substrates out of bacterial cells.

The cryo-EM structures of AdeB-Et indicate that this pump also forms an asymmetric trimer. Surprisingly, in the AdeB-Et-I structure, the periplasmic cleft of one of the AdeB protomers is open, presenting the conformation of the binding state. However, the other two AdeB protomers are more or less identical in conformation, representing the extrusion transient state with the periplasmic clefts closed. These structural features are distinct from the typical conformation of the HAE-RND asymmetric trimeric pumps, including AcrB (34) and MtrD (36), where the three protomers display the binding, access, and extrusion forms with two periplasmic clefts open and one cleft closed.

Surprisingly, the structure of AdeB-Et-I also indicates that a single protomer of AdeB can simultaneously accommodate three bound Et molecules. These three Et ligands are bound at the entrance, distal, and hydrophobic patch drug-binding sites within this binding protomer, suggesting that the plasticity and flexibility of the pump accommodate the simultaneous binding of a number of ligands in a single AdeB protomer. It appears that the Et molecules line the path for drug extrusion, providing us with important information regarding the mechanism of drug export. It is obvious that a drug molecule enters the cleft of the AdeB binding protomer via the periplasmic entrance drug-binding site. The drug molecule will then pass through the proximal drug-binding site, traverse the G-loop, and reach the distal drug-binding site. Notably, the subsequent step is that the bound drug will arrive at the hydrophobic patch binding site, where this site is mostly hydrophobic in nature and mainly composed of aromatic residues, including F178, F277, and W610. Based on the structural information, it is likely that the drug molecule is needed to pass through the hydrophobic patch binding site before being evacuated from the AdeB pump.

Interestingly, the asymmetric trimeric structure of AdeB-Et-II resembles the conformation of the CmeB trimer (35), where the three protomers display resting, extrusion, and binding states, respectively. Again, we found that the binding protomer can accommodate multiple Et ligands simultaneously.

Unexpectedly, in the AdeB-Et-III complex, both the access* and binding protomers were occupied by Et ligands. This observation highlights a phenomenon that individual protomers within the AdeB trimer are capable of independently binding and exporting substrates, as indicated in the case of the C. jejuni CmeB multidrug efflux pump (35). Based on the X-ray structures of ligand-bound RND pumps, including those for AcrB (31, 38) and CusA (39, 40), it is possible that individual protomers of these trimeric pumps can bind substrates simultaneously.

**FIG 6:**
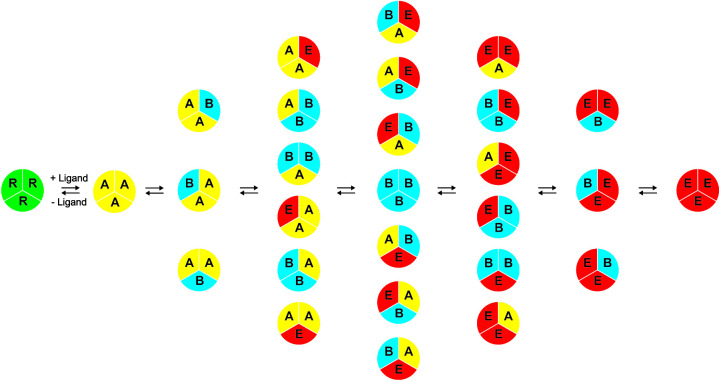
Proposed model of drug efflux mechanism. During drug export, each protomer of the trimeric AdeB pump autonomously undergoes a sequence of conformational transitions. This schematic diagram indicates that each protomer within the AdeB trimer can independently go through a sequence of conformational transitions that lead to the extrusion of substrate (R, resting state; A, access state; B, binding state; E, extrusion state).

Previously, a single-molecule fluorescence resonance energy transfer (FRET) imaging approach was used to study the functional transport dynamics of the C. jejuni CmeB trimeric efflux pump (35). It was observed that each CmeB protomer went through at least four different transient conformations within the transport cycle, and each CmeB protomer within the trimer was able to function independently. In the current work, we observe various conformational states, including the resting, access*, binding, and extrusion forms of the AdeB trimer, using the cryo-EM approach. Our findings also lead us to propose a model for the AdeB transport mechanism (Fig. 6). These experimental data are indeed in good agreement with the single-molecule FRET study of the CmeB pump (35) where each protomer goes through several transient states to open and close the periplasmic cleft accordingly to advance the transport cycle.

## MATERIALS AND METHODS

### Expression and purification of AdeB.

The procedures for cloning, expression, and purification of the A. baumannii (AB0057) AdeB multidrug efflux pump were identical to what was described previously (41). The purified AdeB membrane protein was concentrated to 10 mg/ml in a buffer containing 20 mM Na-HEPES (pH 7.5) and 0.05% (wt/vol) *n*-dodecyl-β-d-maltoside (DDM).

### Fluorescence polarization assay.

The protocols of using fluorescence polarization to quantify *K_D_*s of Et and R6G with AdeB were the same as those described for the AcrB multidrug efflux pump (26). The titration experiments were repeated four times to obtain the average *K_D_* value. Curve fitting was accomplished using Origin (OriginLab Corporation, Northampton, MA).

### Ethidium bromide uptake.

Cells from frozen stocks were inoculated into LB medium containing appropriate antibiotics and incubated for 16 h at 37°C. Cells were then subcultured into 30 ml of fresh LB medium and grown at 37°C to an optical density at 600 nm (OD_600_) of 0.3. The cells then were induced with 1% arabinose for both AbΔ3-pAdeABC and AbΔ3-Pore(pAdeABC) cells. Cells were grown until the OD_600_ was 1.0. Cells were collected by centrifugation at 3,266 × *g* for 30 min at room temperature, and the pellet was washed in 25 ml of 50 mM HEPES-KOH buffer (pH 7.0) containing 1 mM MgSO_4_ and 0.4 mM glucose (HMG buffer) and pelleted at 3,266 × *g* at room temperature. The pellet was resuspended in HMG buffer, adjusted to an OD_600_ of 1.0, and kept at room temperature during the experiment.

An uptake assay was performed in a temperature-controlled multimode microplate reader (Tecan Spark 10M) equipped with a sample injector in fluorescence mode. The fluorescence of Et was monitored at an excitation wavelength of 480 nm and emission wavelength of 610 nm at a gain of 75 for 10 min, as described earlier (42). All measurements were done in duplicates and repeated at least three times.

### Nanodisc preparation.

To assemble AdeB into nanodiscs, a mixture containing 20 μM AdeB, 45 μM MSP (1E3D1), and 930 μM E. coli total extract lipid was incubated for 15 min at room temperature, and then 0.8 mg/ml prewashed Bio-beads (Bio-Rad) was added. The resultant mixture was incubated for 1 h on ice, followed by overnight incubation at 4°C. The protein-nanodisc solution was filtered through 0.22-μm nitrocellulose filter tubes to remove the Bio-beads. To separate free nanodiscs from AdeB-loaded nanodiscs, the filtered protein-nanodisc solution was purified using a Superose 6 column (GE Healthcare) equilibrated with 20 mM Tris-HCl, pH 7.5, and 100 mM NaCl. Fractions corresponding to the size of the trimeric AdeB-nanodisc complex were collected for cryo-EM sample preparation.

### Electron microscopy sample preparation.

A 2 μM AdeB-nanodisc sample was incubated with 20 μM Et for 2 h to form the AdeB-Et complex. The sample was then applied to lysine-coated graphene-oxide grids (Quantifoil Cu R1.2/1.3, 300 mesh) prepared in-house, blotted for 3 s, and then plunge-frozen in liquid ethane using a Vitrobot (Thermo Fisher). The grids were then transferred into cartridges. The images were recorded at −1 to −2.0 μm defocus on a Titan Krios equipped with a K3 summit direct electron detector (Gatan) with counting mode at nominal ×81,000 magnification, corresponding to a sampling interval of 1.08 Å/pixel (superresolution, 0.54 Å/pixel). Each micrograph was exposed for 2.6 s with 18.02 e−/s/physical pixel dose rate (total specimen dose, 40 e−/Å^2^), and 40 frames were captured per specimen area using Latitude.

### Data collection and processing.

The superresolution image stack was aligned, and contrast transfer function (CTF) was estimated using cryoSPARC (43). After manual inspection and sorting to discard poor images, 150 micrographs were picked via blob picker, and particles were classified to generate templates for automated template picking. Initially, 2,188,223 particles were selected after autopicking in cryoSPARC (43). Several iterative rounds of 2D classifications were performed to remove false picks and classes with unclear features, ice contamination, or carbon. The resulting 982,491 particles were further cleaned using *ab initio* and heterogeneous three-dimensional (3D) classification. Particles were extracted to full resolution. A single round of nonuniform refinement followed by local refinement with nonuniform sampling resulted in a consensus structure (see Fig. S2 in the supplemental material).

The consensus structure was then analyzed using 3D variability analysis (3DVA) in cryoSPARC (43). All 3DVA jobs were performed using four modes with a 5-Å filtered resolution. Initially, 3DVA was performed on the full AdeB trimeric protein, resulting in clear opening and closing states of the periplasmic cleft. To separate different conformations of these particles, the periplasmic domain of the AdeB trimer was masked and analyzed using 3DVA with identical settings. The results were clustered and separated into two classes based on the state of the periplasmic clefts and taken for further processing.

The trimeric AdeB particles with three closed periplasmic clefts were subjected to another round of 3DVA by masking the full trimeric protein. The results showed three distinct conformations at the transmembrane region of the pump. Particles were separated and cleaned using one more round of 3DVA. The resulting three distinct structures (AdeB-I, AdeB-II, and AdeB-III) were then refined using nonuniform refinement followed by local refinement with nonuniform sampling, resulting in global resolution maps of 3.64 Å for AdeB-I, 3.21 Å for AdeB-II, and 3.42 Å for AdeB-III based on the gold standard Fourier shell correlation (GS-FSC; 0.143). These maps were then processed using density modification (44) implemented in PHENIX (45), further improving the resolutions to 3.59 Å, 3.13 Å, and 3.27 Å for AdeB-I, AdeB-II, and AdeB-III, respectively (Fig. S2).

The trimeric AdeB particles with one or two open periplasmic clefts were subjected to classification using 3DVA, focusing on the periplasmic region. After classification, particles were further cleaned using 3DVA clustering. The resulting classes were refined using nonuniform refinement followed by local refinement with nonuniform sampling. The results gave rise to global resolution maps of 2.97 Å for AdeB-Et-I, 3.79 Å for AdeB-Et-II, and 3.34 Å for AdeB-Et-III based on the GS-FSC (0.143). These maps were then processed using density modification (44) implemented in PHENIX (45), further improving the resolutions to 2.96 Å, 3.55 Å, and 3.21 Å for AdeB-Et-I, AdeB-Et-II, and AdeB-Et-III, respectively (Fig. S2).

### Model building and refinement.

Model buildings of AdeB-I, AdeB-II, AdeB-III, AdeB-Et-I, AdeB-Et-II, and AdeB-Et-III were based on the 3.59-Å, 3.13-Å, 3.27-Å, 2.96-Å, 3.55-Å, and 3.21-Å cryo-EM maps, respectively. The structure of AdeB (PDB entry 6OWS) (41) was fit into the density maps as a starting model using Chimera (46). The subsequent model rebuilding was performed using Coot (47). Structural refinements were performed using the phenix.real_space_refine program (48) from the PHENIX suite (45). The final atomic model was evaluated using MolProbity (49). The statistics associated with data collection, 3D reconstruction, and model refinement are included in Table S1.

### MD simulations.

The protonation states of the titratable residues of the AdeB pump were determined using the H++ server (http://biophysics.cs.vt.edu/). We used the binding protomer (AdeB [Et]) and one of the extrusion protomers (AdeB [apo]) of the cryo-EM structure of the AdeB-Et-I complex as the template. These two structures were separately immersed in an explicit lipid bilayer consisting of POPC and POPE with a molecular ratio of 1:1 and a water box with dimensions of 152.7 Å by 152.7 Å by 163.6 Å using the CHARMM-GUI Membrane Builder webserver (http://www.charmm-gui.org/?doc=input/membrane). We then added 150 mM NaCl and extra neutralizing counter ions for the simulations. The total numbers of atoms were 281,283 and 278,953 for the AdeB (apo) and AdeB (Et) systems, respectively. The Antechamber module of AmberTools was employed to generate parameters for Et by using the general AMBER force field (GAFF) (50, 51). The partial charges of Et were calculated using *ab initio* quantum chemistry at the HF/6-31G* level (GAUSSIAN 16 program) (Gaussian Inc., Wallingford, CT). The RESP charge-fitting scheme was used to calculate partial charges on the atoms (52). The PMEMD.CUDA program implemented in AMBER18 (UCSF) was used to conduct MD simulations. The simulations were performed with periodic boundary conditions to produce isothermal-isobaric ensembles. Long-range electrostatics were calculated using the particle mesh Ewald (PME) method (53) with a 10-Å cutoff. Prior to the calculations, energy minimization of these systems was carried out. Subsequently, the systems were heated from 0 K to 303 K using Langevin dynamics with a collision frequency of 1 ps^−1^. During heating, the AdeB protomer was position restrained using an initial constant force of 500 kcal/mol/Å^2^ and weakened to 10 kcal/mol/Å^2^ to allow for the movement of lipid and water molecules. The systems then went through 5-ns equilibrium MD simulations. Finally, 1-μs production MD simulations were conducted. During simulations, the coordinates were saved every 100 ps for analysis. GROMCAS analysis tools were used for the MD simulation trajectory analysis (54).

### TMD simulations.

Target MD (TMD) was performed using the NAMD program (55) with the AMBER force field parameters described above. In the simulations, we selected the heavy atoms of the Et ligand at the entrance binding site to be guided toward the target position at the hydrophobic patch-binding site by the application of steering forces. The root mean square (RMS) distance between the current coordinates and the target structure was calculated at each time step. The force on each selected atom was given by a gradient of potential as a function of the RMS values. The system went through energy minimization, heating, and 15-ns equilibrium MD simulations. TMD simulation then was performed for 5 ns based on the MD equilibrated coordinates. A value of 500 kcal/mol/Å^2^ was used as an elastic constant for TMD forces during the simulations.

### Data availability.

Atomic coordinates and density maps have been deposited under accession codes 7KGD (PDB) and EMD-22866 (EMDB) for AdeB-I, 7KGE (PDB) and EMD-22867 (EMDB) for AdeB-II, 7KGF (PDB) and EMD-22868 (EMDB) for AdeB-III, 7KGG (PDB) and EMD-22869 (EMDB) for AdeB-Et-I, 7KGH (PDB) and EMD-22870 (EMDB) for AdeB-Et-II, and 7KGI (PDB) and EMD-22871 (EMDB) for AdeB-Et-III.
