# Supplementary material for: Cryoelectron Microscopy Structures of AdeB Illuminate Mechanisms of Simultaneous Binding and Exporting of Substrates
Source: mBio. 2021 Feb 23;12(1):e03690-20. doi: 10.1128/mBio.03690-20 (PMC8545137; doi:10.1128/mBio.03690-20)
Supplement: FIG S1 [file mbio.03690-20-sf001.pdf]

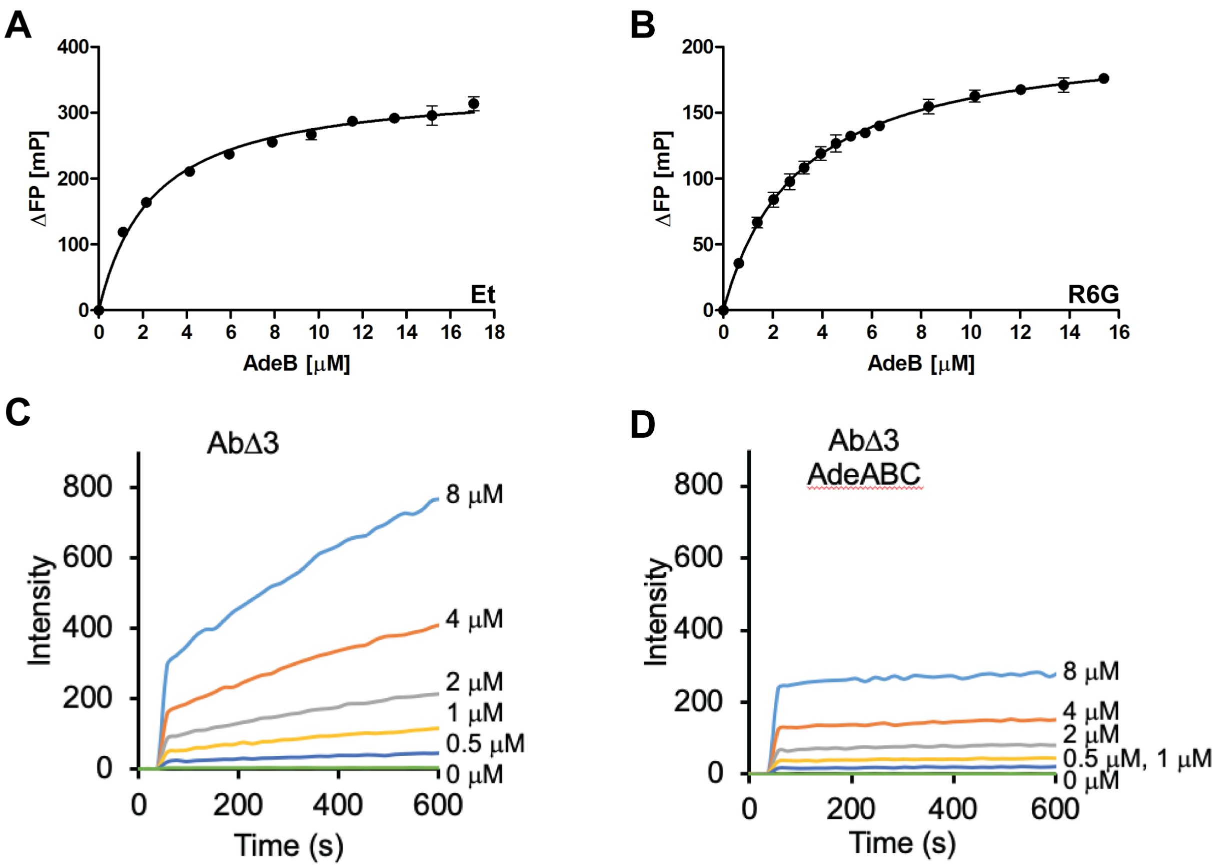

Figure S1. AdeB and Ethidium interactions. (A) Fluorescence polarization (FP) assay shows Et binds to AdeB with an affinity of 2.5 ± 0.1 μM. (B) FP shows R6G binds AdeB with an affinity of 3.1 ± 0.1 μM. (C) Kinetics of intracellular accumulation of Et in the *A. baumannii* AbΔ3 cells lacking the AdeABC, AdeIJK and AdeFGH efflux pumps. Et accumulates inside of cells with all three pump systems are knocked out (green, 0 μM Et; blue, 0.5 μM Et; yellow, 1 μM Et; gray, 2 μM Et; red, 4 μM Et; and cyan, 8 μM Et). (D) Complementing triple knockout cells with AdeABC restores extrusion and halts accumulation of Et inside of cells (green, 0 μM Et; blue, 0.5 μM Et; yellow, 1 μM Et; gray, 2 μM Et; red, 4 μM Et; and cyan, 8 μM Et).
