# Supplementary material for: Cryoelectron Microscopy Structures of AdeB Illuminate Mechanisms of Simultaneous Binding and Exporting of Substrates
Source: mBio. 2021 Feb 23;12(1):e03690-20. doi: 10.1128/mBio.03690-20 (PMC8545137; doi:10.1128/mBio.03690-20)
Supplement: FIG S2 [file mbio.03690-20-sf002.pdf]

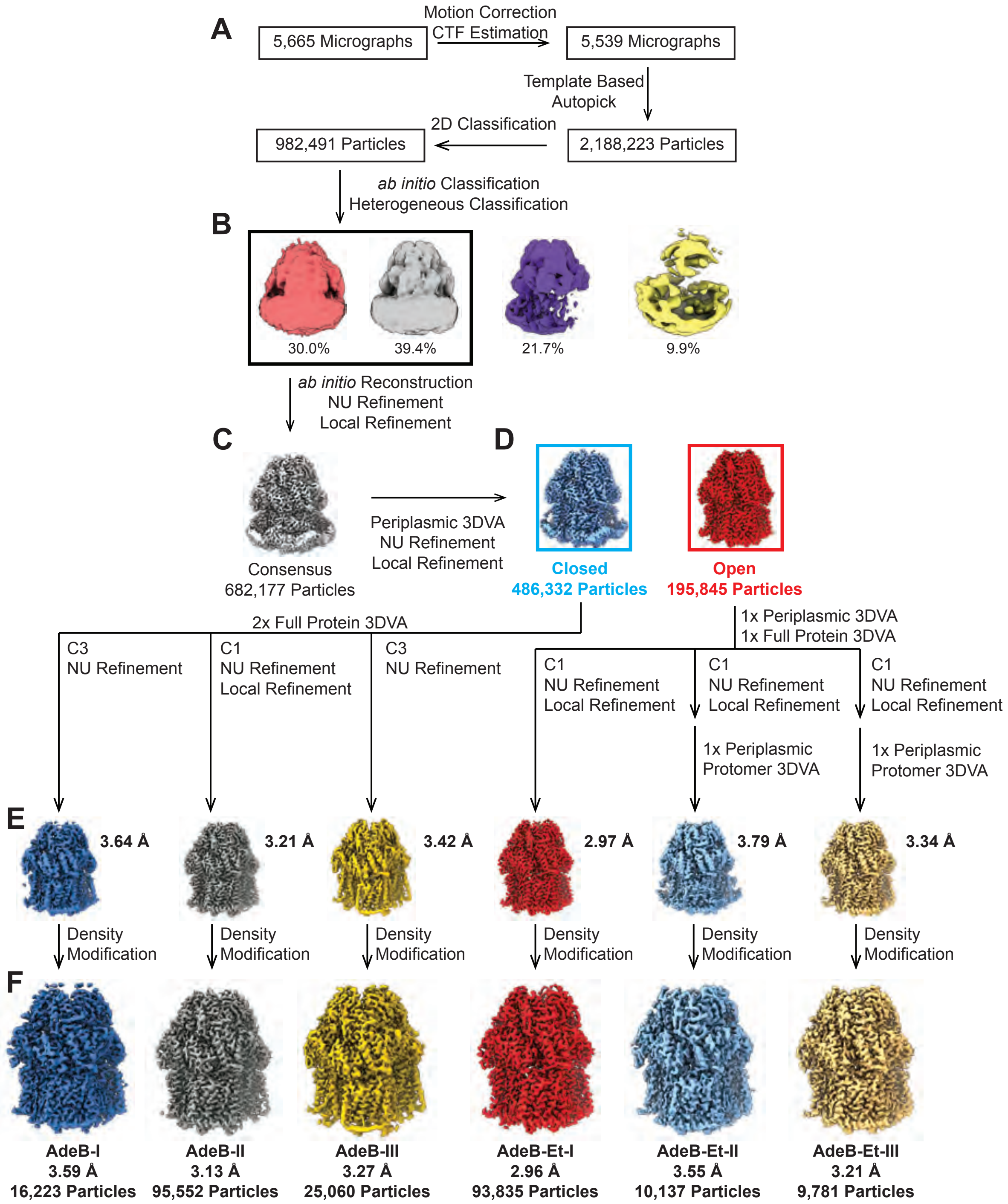


**Figure S2. AdeB Processing Workflow.** (A) Processing of 5,665 micrographs to get initial pool of 982,491 particles. (B) 3D classification results. (C) Two 3D classes gave rise to consensus refinement with 682,177 total particles. (D) Focused classification of only the periplasmic domains using 3D variability analysis (3DVA) gave rise to two different populations, the apo form (protomers closed) and the bound form (one or two protomers open). (E) Further classification and cleaning by 3DVA gave rise to six total structures (3 apo and 3 bound) with marked differences. (F) Final density modified maps and resolutions.
