# Supplementary material for: Cryoelectron Microscopy Structures of AdeB Illuminate Mechanisms of Simultaneous Binding and Exporting of Substrates
Source: mBio. 2021 Feb 23;12(1):e03690-20. doi: 10.1128/mBio.03690-20 (PMC8545137; doi:10.1128/mBio.03690-20)
Supplement: FIG S3 [file mbio.03690-20-sf003.pdf]

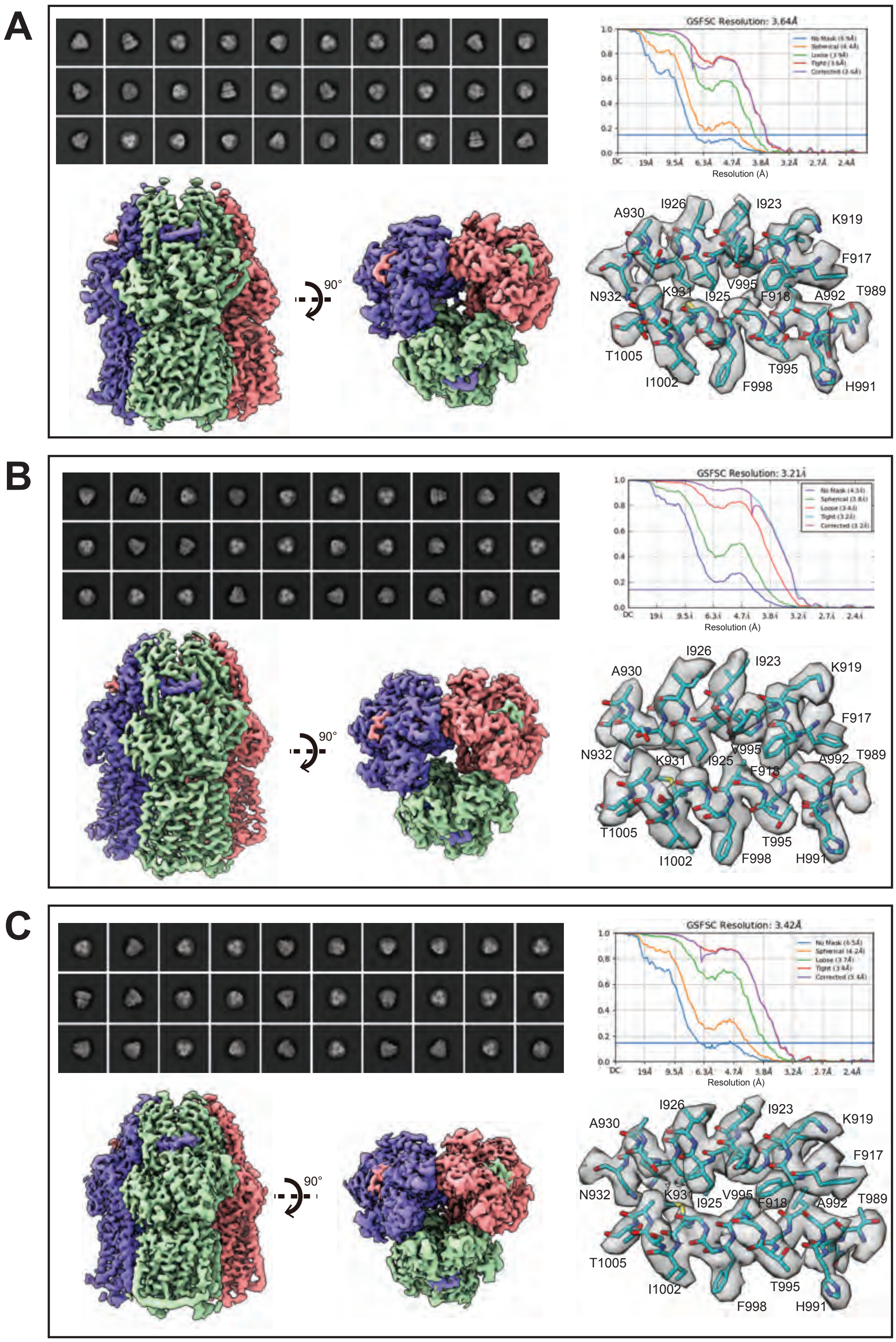

Figure S3. Cryo-EM analysis of apo AdeB. (A) AdeB-I cryo-EM results. (B) AdeB-II cryo-EM results. (C) AdeB-III cryo-EM results. Panels depict representative 2D classes (top left), GS-FSC resolution curve (top right), final cryo-EM map (bottom left) and representative density (bottom right) for each structure. Amino acids are represented as cyan sticks.
