# Supplementary material for: Cryoelectron Microscopy Structures of AdeB Illuminate Mechanisms of Simultaneous Binding and Exporting of Substrates
Source: mBio. 2021 Feb 23;12(1):e03690-20. doi: 10.1128/mBio.03690-20 (PMC8545137; doi:10.1128/mBio.03690-20)
Supplement: FIG S4 [file mbio.03690-20-sf004.pdf]

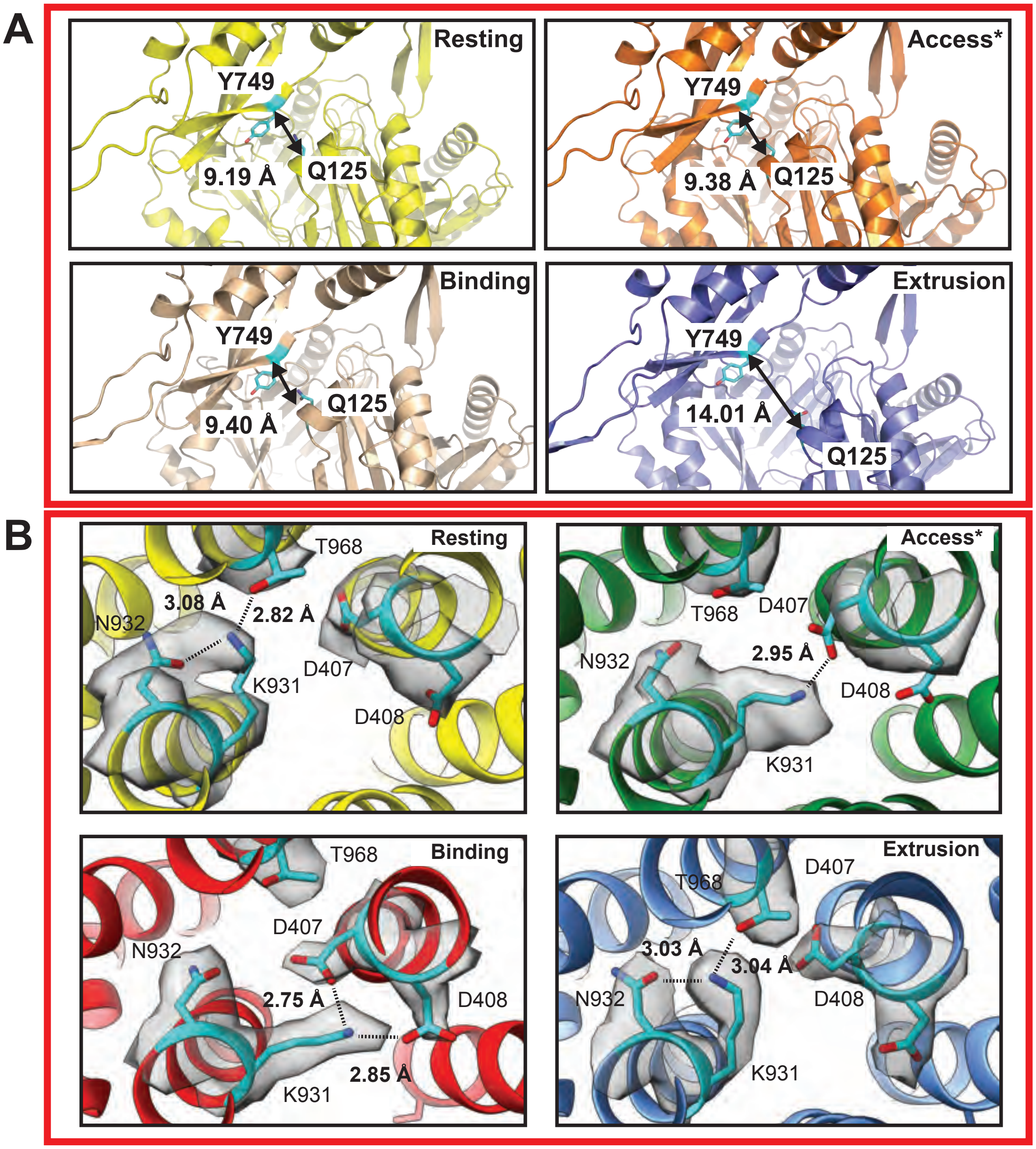

Figure S4. AdeB Protomer Classification. (A) Example extrusion tunnel measurements for resting (top left), access* (top right), binding (bottom left) and extrusion (bottom right) states. In the resting (AdeB-Et-II chain A), access* (AdeB-Et-III chain A) and binding (AdeB-Et-I chain C) states, the extrusion tunnel is closed with distances of 9.19 Å, 9.38 Å and 9.40 Å, respectively, between the Cα atoms of Q125 and Y749. In the extrusion protomer (AdeB-Et-I chain B), the extrusion tunnel is open with a measured distance of 14.01 Å between Q125 and Y749 allowing export of ligand. (B) Proton-relay network measurements. In the resting protomer (AdeB-Et-II chain A, top left), the NZ atom of K931 is within hydrogen bonding distance of the O atoms of N932 and T968. In the access* protomer (AdeB-Et-III chain A, top right), K931 swings away from N932 and T968 to interact with D407. In the binding protomer (AdeB-Et-I chain C, bottom left), NZ of K931 interacts with the O atoms of D407 and D408. In the extrusion protomer (AdeB-II chain B, bottom right), K931 swings back to interact with N932 and T968, where NZ of K931 is within hydrogen bonding distance of O atoms on N932 and T968.
