# Supplementary material for: Cryoelectron Microscopy Structures of AdeB Illuminate Mechanisms of Simultaneous Binding and Exporting of Substrates
Source: mBio. 2021 Feb 23;12(1):e03690-20. doi: 10.1128/mBio.03690-20 (PMC8545137; doi:10.1128/mBio.03690-20)
Supplement: TABLE S1 [file mbio.03690-20-st001.pdf]

Table S1. Cryo-EM data collection and refinement statistics.

**Data collection**						
Magnification	81,000					
Voltage (kV)	300					
Electron Microscope	Krios-GIF-K3					
Defocus (um)	-1.0 to -2.0					
Total exposure time (s)	2.6					
Energy filter width (eV)	20					
Pixel size (Å)	1.08 (0.54)					
Total dose (e^-/ Å^2)	40					
Number of frames	40					
Does rate (e^-/ Å^2/phys. Pixel)	18.02					
Number of micrographs	5,669					
Initial particle images (no.)	2,188,223					
Final particle images (no.)	982,491					
**Refinement**	**AdeB-I**	**AdeB-II**	**AdeB-III**	**AdeB-Et-I**	**AdeB-Et-II**	**AdeB-Et-III**
Total Particles (no.)	16,223	95,552	25,060	93,835	10,137	9,781
GS-FSC Resolution (0.143, Å)	3.64	3.21	3.42	2.97	3.79	3.34
FSC-model Resolution after Density Modification (0.5, Å)	3.59	3.13	3.27	2.96	3.55	3.21
Symmetry	C3	C1	C3	C1	C1	C1
Model composition						
Protein residues	3,060	3,060	3,060	3,060	3,060	3,060
Ligands	6	3	0	9	5	6
RMSD						
Bond lengths (Å)	0.006	0.005	0.006	0.006	0.005	0.005
Bond angles (°)	1.222	1.058	1.110	1.115	1.079	1.065
**Validation**	**AdeB-I**	**AdeB-II**	**AdeB-III**	**AdeB-Et-I**	**AdeB-Et-II**	**AdeB-Et-III**
MolProbity score	1.70	1.37	1.59	1.25	1.49	1.40
Clash score	9.40	6.66	9.04	4.37	9.15	7.35
Ramachandran plot						
Favored (%)	97.87	98.56	97.44	99.05	98.26	98.06
Allowed (%)	2.13	1.44	2.56	0.95	1.74	1.94
Disallowed (%)	0.00	0.00	0.06	0.00	0.00	0.00
CC Mask	0.71	0.79	0.74	0.82	0.72	0.78
