# Supplementary material for: Cryoelectron Microscopy Structures of AdeB Illuminate Mechanisms of Simultaneous Binding and Exporting of Substrates
Source: mBio. 2021 Feb 23;12(1):e03690-20. doi: 10.1128/mBio.03690-20 (PMC8545137; doi:10.1128/mBio.03690-20)
Supplement: TABLE S2 [file mbio.03690-20-st002.pdf]

Table S2. Different transient states of the AdeB protomers.

Protomer	Cleft State	Exit site distance, Q125 to Y749	Hydrogen-bonded distance, K931 to				Protomer Assignment
			D407 (Å)	D408 (Å)	N932 (Å)	T968 (Å)	
AdeB-I, A	Closed	13.04	-	-	3.00	3.15	Extrusion
AdeB-I, B	Closed	13.04	-	-	3.00	3.15	Extrusion
AdeB-I, C	Closed	13.04	-	-	3.00	3.15	Extrusion
AdeB-II, A	Closed	13.53	-	-	3.12	3.17	Extrusion
AdeB-II, B	Closed	14.89	-	-	3.03	3.04	Extrusion
AdeB-II, C	Closed	13.99	-	-	3.15	3.00	Extrusion
AdeB-III, A	Closed	14.24	-	-	3.20	3.01	Extrusion
AdeB-III, B	Closed	14.24	-	-	3.20	3.01	Extrusion
AdeB-III, C	Closed	14.24	-	-	3.20	3.01	Extrusion
AdeB-Et-I, A	Closed	13.87	-	-	3.17	2.86	Extrusion
AdeB-Et-I, B	Closed	14.01	-	-	3.03	3.18	Extrusion
AdeB-Et-I, C	Open	9.40	2.75	2.85	-	-	Binding
AdeB-Et-II, A	Closed	9.19	-	-	3.08	2.82	Resting
AdeB-Et-II, B	Closed	15.25	-	-	2.79	2.71	Extrusion
AdeB-Et-II, C	Open	9.01	3.12	2.67	-	-	Binding
AdeB-Et-III, A	Open	9.38	2.95	-	-	-	Access*
AdeB-Et-III, B	Closed	14.71	-	-	2.62	2.99	Extrusion
AdeB-Et-III, C	Open	8.50	2.93	2.97	-	-	Binding

Protomer states were defined using three criteria. (1) State of the periplasmic cleft between PC1 and PC2. (2) Size of the extrusion tunnel measured by the distance between the Cα atoms of Q125 and Y749, which form the substrate exit site. (3) Hydrogen-bonded distance between the “proton sweeper” K931 and other residues within the proton-relay network (D407, D408, N932 or T968). Only distances of 3.20 Å and under are noted.
