# Supplementary material for: Cryoelectron Microscopy Structures of AdeB Illuminate Mechanisms of Simultaneous Binding and Exporting of Substrates
Source: mBio. 2021 Feb 23;12(1):e03690-20. doi: 10.1128/mBio.03690-20 (PMC8545137; doi:10.1128/mBio.03690-20)
Supplement: FIG S5 [file mbio.03690-20-sf005.pdf]

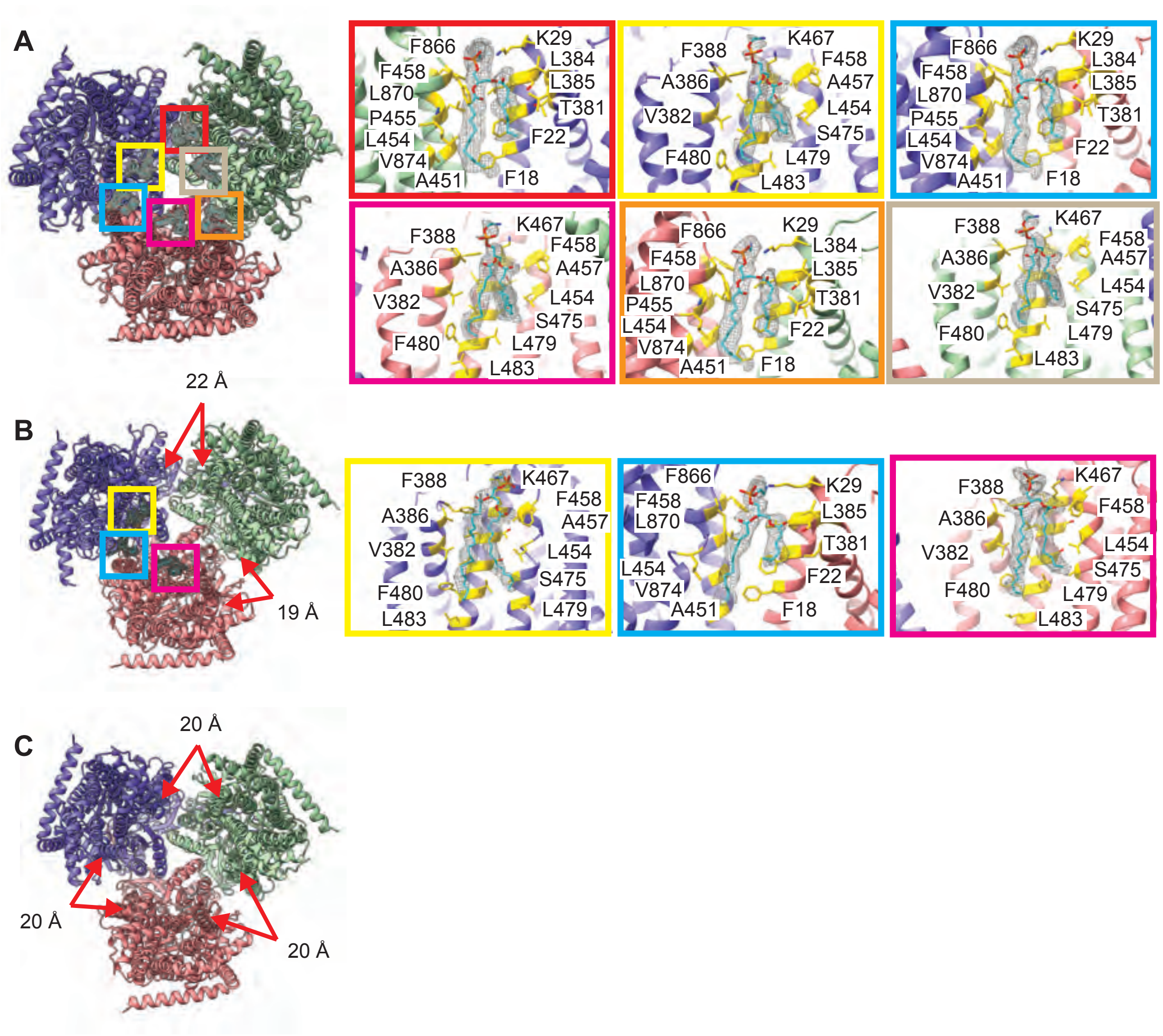

Figure S5. Lipid binding sites. (A) AdeB-I has six bound lipids in the transmembrane domain, one between each protomer and another on each individual protomer. Colored boxes correspond to blown up view. (B) AdeB-II has three bound lipids in the transmembrane domain, one between the purple and pink protomers, one on the purple protomer and another on the pink protomer. It appears that the green protomer, which does not interact with the purple and pink protomer at the transmembrane region, does not have any bound lipids. (C) No lipids are present at the transmembrane domain of AdeB-III. The three protomers are 20 Å away from each other as measured between V10 and A881 Cα atoms.
