# Supplementary material for: Cryoelectron Microscopy Structures of AdeB Illuminate Mechanisms of Simultaneous Binding and Exporting of Substrates
Source: mBio. 2021 Feb 23;12(1):e03690-20. doi: 10.1128/mBio.03690-20 (PMC8545137; doi:10.1128/mBio.03690-20)
Supplement: FIG S7 [file mbio.03690-20-sf007.pdf]

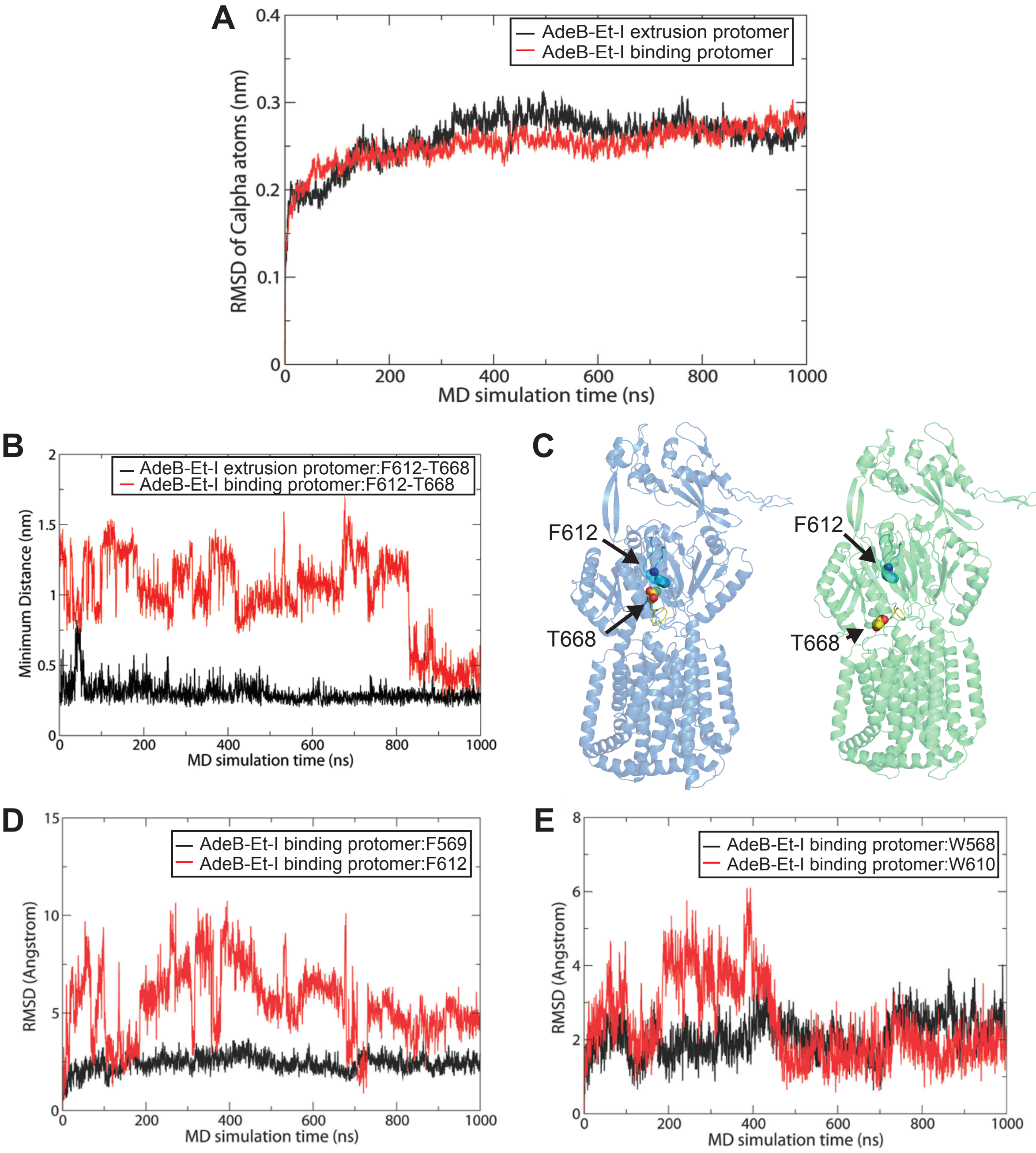

Figure S7. Results of 1 μs Simulation. (A) MD simulation results of the AdeB-Et-I extrusion protomer (black), and AdeB-Et-I binding protomer (red). The Cα atoms RMSD (root mean square deviation) are based on the MD simulation trajectories (1 μs). (B) Distances between residues F612 and T668 over the course of the simulation (1 μs) for both extrusion (black) and binding (red) protomers from AdeB-Et-I. (C) The G-loop (cyan) and F-loop (yellow) form a gate, which is closed in the extrusion protomer (blue cartoon). Residues F612 (cyan spheres) and T668 (yellow spheres) interact through hydrophobic interactions to close the gate. In the binding protomer (green cartoon), the F- and G-loops separate to allow ligand entry. (D) RMSD comparison of F612 and nearby residue F569 over the 1 μs simulation. (E) RMSD comparison of W610 and nearby residue W568. W610 and F612 are critical for the transport of ligand out of the pump.
