# Supplementary material for: Cryoelectron Microscopy Structures of AdeB Illuminate Mechanisms of Simultaneous Binding and Exporting of Substrates
Source: mBio. 2021 Feb 23;12(1):e03690-20. doi: 10.1128/mBio.03690-20 (PMC8545137; doi:10.1128/mBio.03690-20)
Supplement: FIG S8 [file mbio.03690-20-sf008.pdf]

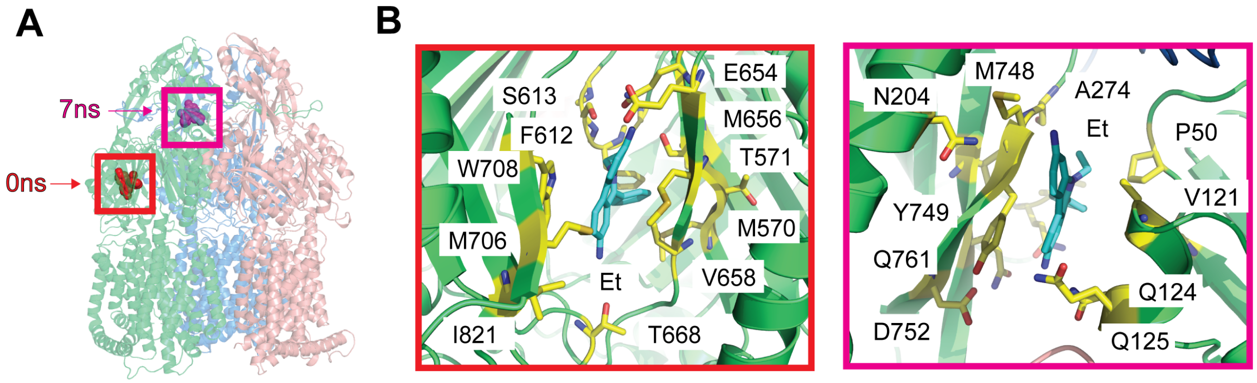


**Figure S8. Targeted MD Simulation. (**A) Highlighted timepoints for focus on interactions between Et and AdeB (0 ns and 7 ns). Box colors correspond to blown-up images. (B) Focused images of AdeB-Et interactions at selected timepoints from TMD simulation. Locations correspond to the entrance binding site (red) and ethidium leaving through the extrusion tunnel (magenta). Et is colored cyan while all residues within 4.5 Å are highlighted with yellow sticks in all images.
